# Investigation of ovarian cancer associated sialylation changes in N-linked glycopeptides by quantitative proteomics

**DOI:** 10.1186/1559-0275-9-10

**Published:** 2012-08-02

**Authors:** Vivekananda Shetty, Julie Hafner, Punit Shah, Zacharie Nickens, Ramila Philip

**Affiliations:** 1 Immunotope, Inc., 3805 Old Easton Road, Doylestown, PA, 18902, USA

**Keywords:** Ovarian cancer, Quantitative proteomics, Sialylation, Lectin, N-linked glycopeptides, Mass spectrometry, Western blot

## Abstract

**Background:**

In approximately 80% of patients, ovarian cancer is diagnosed when the patient is already in the advanced stages of the disease. CA125 is currently used as the marker for ovarian cancer; however, it lacks specificity and sensitivity for detecting early stage disease. There is a critical unmet need for sensitive and specific routine screening tests for early diagnosis that can reduce ovarian cancer lethality by reliably detecting the disease at its earliest and treatable stages.

**Results:**

In this study, we investigated the N-linked sialylated glycopeptides in serum samples from healthy and ovarian cancer patients using Lectin-directed Tandem Labeling (LTL) and iTRAQ quantitative proteomics methods. We identified 45 N-linked sialylated glycopeptides containing 46 glycosylation sites. Among those, ten sialylated glycopeptides were significantly up-regulated in ovarian cancer patients’ serum samples. LC-MS/MS analysis of the non-glycosylated peptides from the same samples, western blot data using lectin enriched glycoproteins of various ovarian cancer type samples, and PNGase F (+/−) treatment confirmed the sialylation changes in the ovarian cancer samples.

**Conclusion:**

Herein, we demonstrated that several proteins are aberrantly sialylated in N-linked glycopeptides in ovarian cancer and detection of glycopeptides with abnormal sialylation changes may have the potential to serve as biomarkers for ovarian cancer.

## Background

The American Cancer Society estimates that in 2011, about 21,990 new cases of ovarian cancer will be diagnosed and 15,460 women will die of ovarian cancer in the United States (ovariancancer.org) [1-3]. When ovarian cancer is detected early, the five year survival rate is over 90% [4]. Serum measurement of CA125, the current standard, has an early stage detection rate of only about 28% and when combined with ultrasound still only identifies 48% [5,6]. Development of improved diagnostic tools for early detection of ovarian cancer, including the discovery of new ovarian cancer biomarkers, has the potential to significantly improve the survival rate.

It has been shown that in the cancer transformation process, changed expression and post translational modification of proteins occurs, resulting in a change in the protein structure and function. Investigating these modifications specific for cancer may provide vital information and serve as biomarkers for the diseased state. Glycosylation is a common and essential form of post translational modification of proteins. Among all the glycosylation forms, sialylation has received much attention owing to the strong correlation between the sialylation aberration and cancer [7]. Sialic acid residues are known to be linked via an R-2,3 or an R-2,6 bond to Gal/GalNAc in proteins. SNA lectin binds to peptides carrying a sialic acid residue connected to the underlying sugar chains through an R-2,6 linkage. It has been suggested that there is an increased branching of glycan structures in cancer along with the increased expression both at RNA and protein level of sialyltransferase [8-10], which leads to a global increase in sialyation of the proteins [11]. Increased activity of sialyltransferase is also shown to be accompanied by an increase in the level of enzymes, such as ST6Gal-1, which is responsible for linking sialic acid to galactose in colorectal, ovarian and breast cancers [10,12-14]. ST6Gal-1 has been implicated in cell-cell interaction, enhanced motility and increased invasiveness of tumor cells [10].

In the last decade, with the evolution of proteomics and glycomics technologies, the potential for the identification of biomarkers has increased tremendously, in spite of the extreme complexity of the serum with a dynamic range in concentration of several orders of magnitude [15]. In order to identify these low abundant disease marker proteins in serum, various methods have been developed to deplete the abundant proteins such as albumin and IgG, which constitutes about 90% of the serum protein concentration [16,17]. Alternatively, several methods were developed to enrich a specific class of proteins, such as glycoproteins by a lectin affinity enrichment strategy, which can increase the chances for the identification of elusive glycosylation changes in low abundant proteins. Recent advances in glycoproteomics have made it possible to probe specific glycosylation changes [18], in particular sialylation changes [19-21], in proteins between the disease and normal state. The level of sialic acid was observed to be significantly elevated in ovarian cancer patients plasma compared to the healthy controls [19,20]. Berbec et al. [21], reported that the average concentration of sialic acid in total serum in ovarian cancer patients was significantly higher than in the healthy control group and may reflect the development of malignancy and should be considered as a supporting tumor marker in ovarian cancer diagnosis. In recent years, several groups have investigated the sialylation aberration in the glycoproteome of cancer serum samples using diverse proteomics strategies including lectin affinity, hydrazine chemistry, HPLC and chemical enrichment methods [18,22-27].

In our previous work, we probed the prostate cancer serum glycoproteome by employing the lectin-directed tandem labeling (LTL) quantitative proteomics method [28] and identified several N-linked sialylated glycopeptides that showed significant sialylation aberration between normal and prostate cancer serum samples. In the current study, we report the results of the sialylation aberration analysis in ovarian cancer-associated N-linked glycoproteins. We employed the LTL method to identify N-linked sialylation sites and accurately identified the changes in sialylation between normal and ovarian cancer serum samples based on the N-deglycosylated peptide analysis [29,30]. We used SNA lectin to capture sialylated glycopeptides and, for quantitation, we used acetyl (^1^H_3_/^2^D_3_) labeling at the N-terminus in combination with ^18^O labeling during PNGase F digestion for glycosylation site mapping. Further, iTRAQ quantitative analysis of non-glycosylated peptides from the same samples revealed that the observed sialylation changes in cancer serum samples are independent of the glycoprotein concentrations. The quantitative proteomics results were further verified by western blot analysis of SNA enriched selected glycoproteins that are strongly implicated in ovarian cancer.

## Results

### Identification of glycopeptides and determination of glycosylation sites

**Figure 1:**
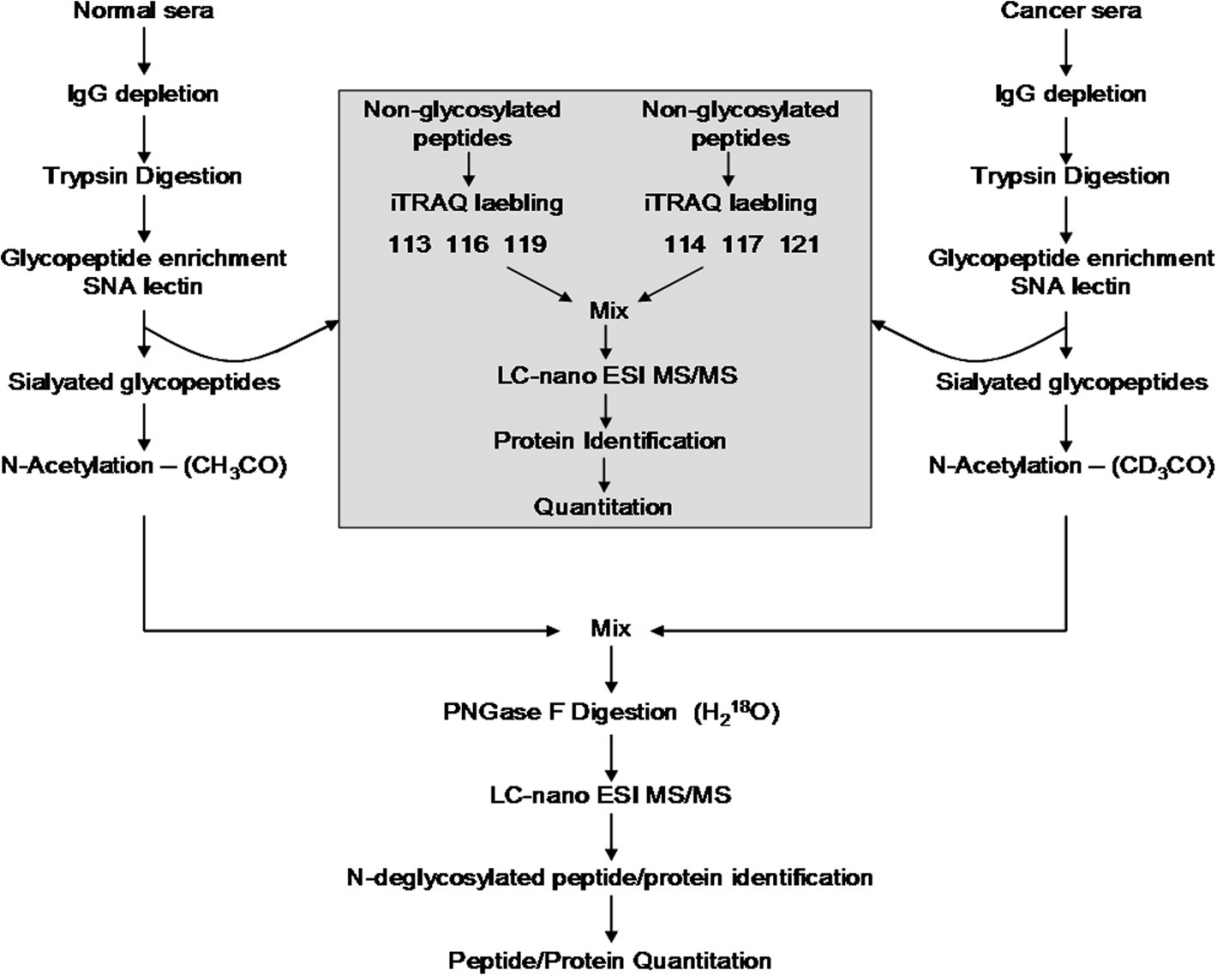
LTL quantitative proteomics strategy used for the identification and quantification of sialylated N-linked glycopeptides in normal and ovarian cancer sera.

**Figure 2:**
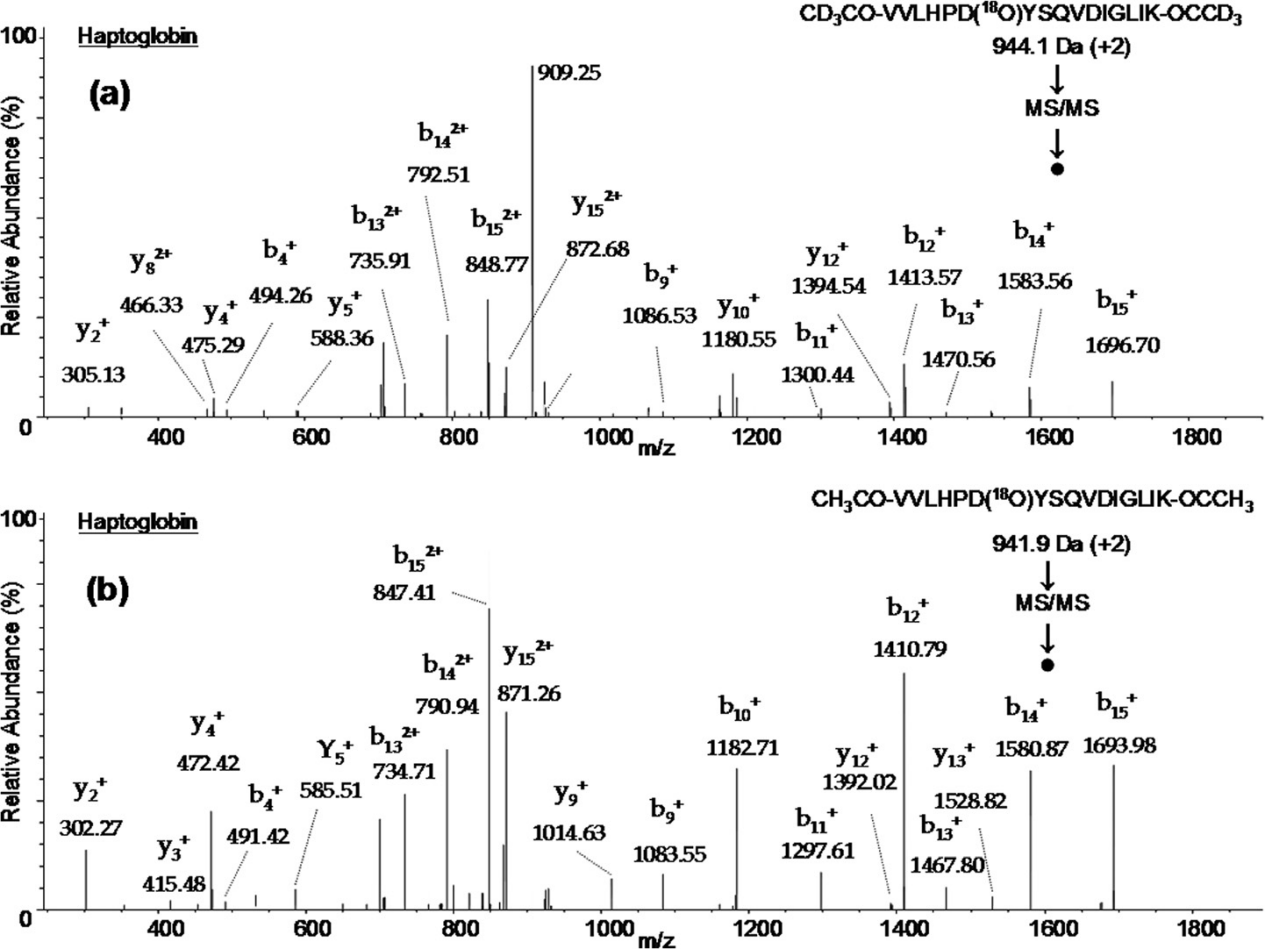
**Tandem mass spectra of doubly charged ions of heavy CD**_**3**_**CO- VVLHPD(**^**18**^**O)YSQVDIGLIK- COCD**_**3**_**and CH**_**3**_**CO- VVLHPD(**^**18**^**O)YSQVDIGLIK- OCCH**_**3**_**peptides as identified by LTL quantitative proteomics in normal and ovarian cancer sera analysis.**

**Figure 3:**
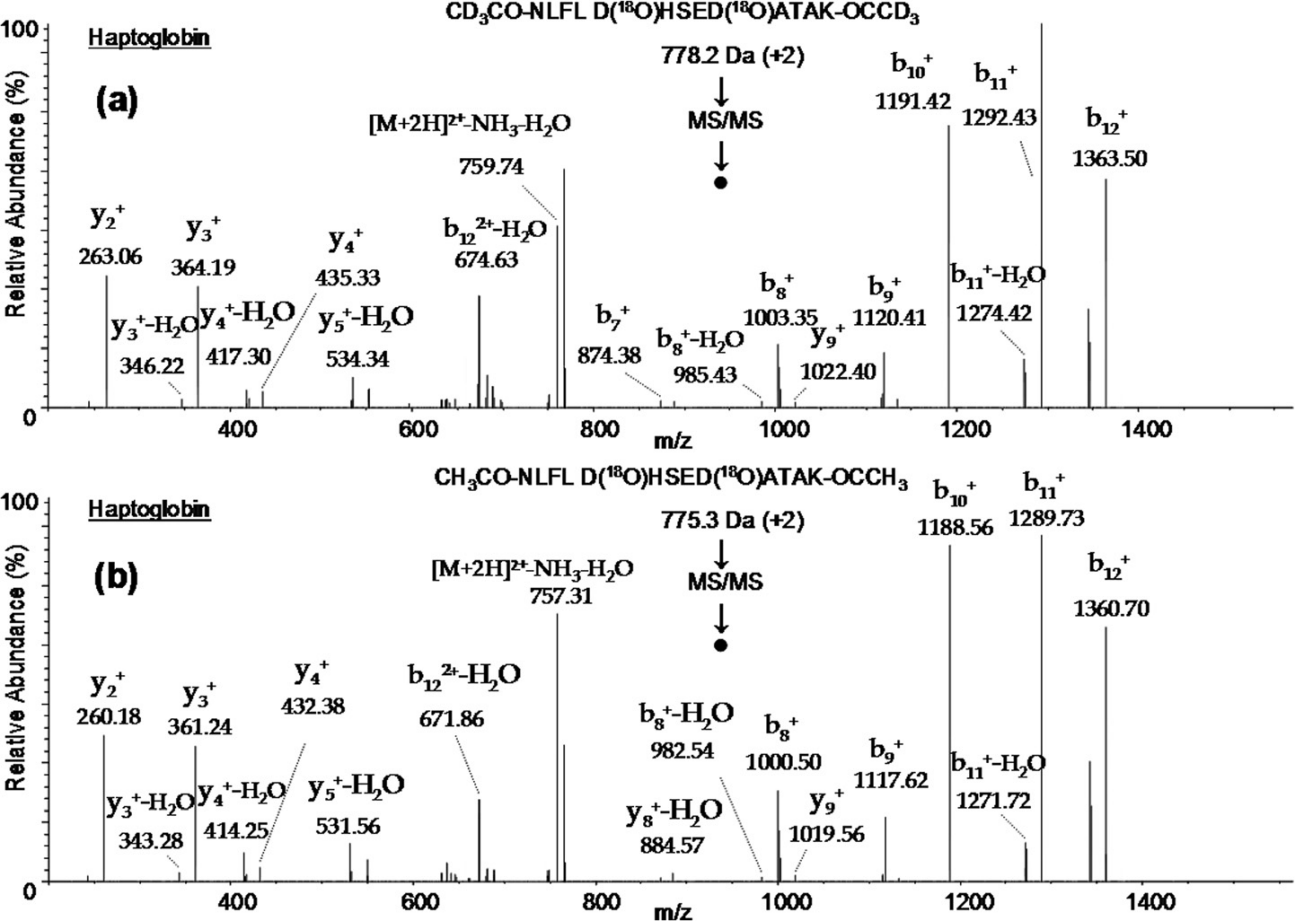
**Tandem mass spectra of doubly charged ions of heavy CD**_**3**_**CO- NLFL D(**^**18**^**O)HSED(**^**18**^**O)ATAK- COCD**_**3**_**and light CH**_**3**_**CO- NLFL D(**^**18**^**O)HSED(**^**18**^**O)ATAK**- **OCCH**_**3**_**peptides as identified by LTL quantitative proteomics in normal and ovarian cancer sera analysis.**

**Figure 4:**
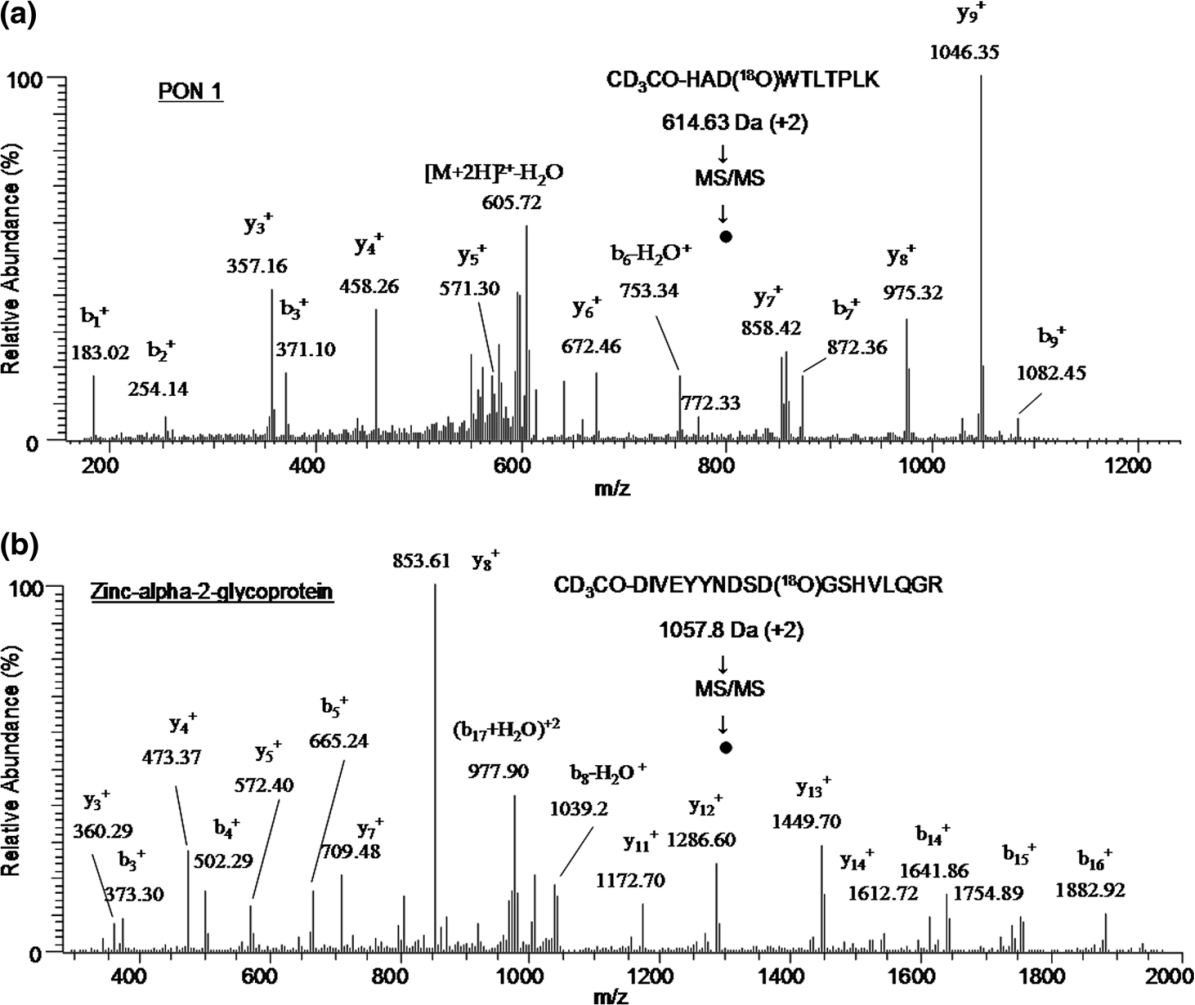
**Tandem mass spectrum of doubly charged ions of (a) heavy CD**_**3**_**CO- HA D(**^**18**^**O)WTLTPLK peptide and (b) CD**_**3**_**CO-DIVEYYNDSD(**^**18**^**O)GSHVLQGR.** Peptide as identified by LTL quantitative proteomics in normal and ovarian cancer sera analysis.

The glycopeptide identification and glycosylation site determination were achieved by following the strategy as outlined in Figure 1. First, abundant IgG glycoproteins were depleted from normal and ovarian cancer patients’ serum samples. Then equal amounts (5 mg each) of protein from normal and cancer serum samples were digested by trypsin and glycopeptides containing sialic acid were enriched by using SNA lectin. The resulting sialylated glycopeptides were labeled using light and heavy isotopes of acetic anhydride reagents and mixed. Next, N-linked glycans were cleaved by PNGase F in the presence of heavy (^18^O) water to introduce a 3 Da mass shift with the aim to unequivocally identify glycosylation sites. Finally, the N-deglycosylated peptide mixture was analyzed by nano LC-MS/MS to identify peptide sequences in addition to determining the glycosylation sites. Each glycopeptide identified in the database search results was inspected for the NXS/T consensus sequence as well as for a 3 Da mass shift. Also, the MS/MS spectrum of each glycopeptide was verified manually and unambiguously characterized the complete peptide sequence. For instance, two glycopeptides (VVLHPNYSQVDIGLIK and NLFLNHSENATAK) were identified for haptoglobin and the tandem mass spectrometry data in Figure 2b shows all the signature ions (b and y ions) confirming the sequence of the light isotope of CH_3_CO-VVLHPD(^18^O)YSQVDIGLIK-OCCH_3_ glycopeptide sequence. Similarly, in the MS/MS spectrum of Figure 2a, all b and y ions, including a shift of 3 Da modification in b ions (b_13_, b_14_, b_15_) and y ions (y_2_, y_4_, y_5_) due to heavy acetyl group at the N-terminus, confirm the identity of the heavy isotope of the CD_3_CO-VVLHPD(^18^O)YSQVDIGLIK-OCCD_3_ glycopeptide. It should be noted that the above two peptides possess NXT/S consensus sequences and, in both peptides, asparagine is modified to aspartic acid, as evidenced by 3 Da mass shift for b_9_ ion in Figure 2a and 2b. However, the total mass is increased by 6 Da from light labeled peptide to heavy labeled peptide because of the acetylation labeling at the N-terminus as well as at the c-terminal lysine side chain. The other haptoglobin glycopeptide, NLFLNHSENATAK, was identified with 2 glycosylation sites and this is the only glycopeptide identified with more than one glycosylation site, as corroborated by its light and heavy labeled peptide tandem mass spectrometry data shown in Figure 3a and 3b, respectively. Similar observations were made for CD_3_CO-HAD(^18^O)WTLTPLK (PON1) and CD_3_CO-DIVEYYnDSD(^18^O)GSHVLQGR (alpha-2-glycoprotein 1, zinc) glycopeptides as confirmed by their MS/MS data (Figure 4). For these two proteins, the light labeled versions of the peptides were not identified in our analysis. The total number of glycopeptides identified in the current study and the relevant details are given in Table 1. In total, we identified 45 glycopeptides derived from 30 sialylated glycoproteins. Overall, we were able to identify 46 glycosylation sites in 45 N-linked glycopeptides and all these sites have been reported in the literature (Swissprot database). The majority of the sialylated glycopeptides identified in the current study were previously reported by various proteomics methods signifying the strength of our LTL quantitative method to investigate glycosylation aberrations and glycosylation sites in proteins in cancer. Furthermore, identification of low abundant serum proteins including PON 1 (25 μg/mL) [31], Ficolin-3 (32.4 μg/mL) [32], and Kallikrein (2.9 μg/mL) [33] in our analysis demonstrate the high sensitivity of the LTL method.

**Table 1:** List of identified glycosylation sites and the results of quantitation of N-deglycosylated peptides

**Swissprot ID**	**Protein**	**Glycopeptide sequence**	**Glycosylation site**	**Charge**	**Observed mass (Da)**	**Molecular weight (Da)**		**Δ mass (Da)**	**Mascot score**	**Heavy/Light Ratio (Peptide)a**	**Mean ratio (Protein)**
						**Mr(expt)**	**Mr(calc)**				
***Proteins with increase in sialylation***											
P01009	**Alpha-1-antitrypsin**^**†**^										2.1
		YLG**NAT**AIFFLPDEGK^*^	271	2	902.29	1802.57	1802.90	-0.33	88	**2.1**	
O75882	Attractin^†^										2.2
		**NHS**CSEGQISIFR^*^	731	2	791.71	1581.41	1581.71	-0.31	54	**2.2**	
**P00738**	Haptoglobin^†^										2.4
		VVLHP**NYS**QVDIGLIK^*^	241	2	941.60	1881.18	1881.01	0.17	**51**	**2.7**	
		NLFL**NHS**E**NAT**AK	207,211	2	775.05	1548.08	1547.72	0.36	68	**2.0**	
P02787	Serotransferrin^†^										5.4
		CGLVPVLAENYNK^*^	432	2	**763.12**	1524.23	1523.76	0.47	88	**7.6**	
		QQQHLFGS**NVT**DCSGNFCLFR^*^	630	2	1281.94	2561.86	2562.13	-0.27	44	**3.1**	
P27169	Serum paraoxonase/arylesterase 1 (PON1)^†^										4.0
		HA**NWT**LTPLK^*^	253	2	614.82	1227.63	1227.65	-0.03	58	**4.0**	
P04114	Apolipoprotein B-100^†^										1.5
		FVEGSH**NST**VSLTTK^*^	3411	2	**827.75**	1653.48	1653.81	**-0.33**	91	**2.0**	
		YDF**NSS**MLYSTAK	3465	2	795.71	1589.41	1589.68	-0.27	68	**1.0**	
P02790	Hemopexin^†^										1.9
		ALPQPQNVTSLLGCTH^*^	453	2	897.77	1793.52	1793.88	-0.36	83	**2.1**	
		SWPAVG**NCS**SALR	187	2	725.38	1448.75	1448.66	0.09	83	**1.6**	
P25311	Zinc-alpha-2-glycoprotein^†^										1.5
		FGCEIEN**NR**	128	2	592.57	1183.14	1182.48	0.65	71	**0.9**	
		DIVEYY**NDS**NGSHVLQGR^*^	109	2	1057.82	2113.63	2112.96	0.67	54	**2.0**	
***Proteins with increase and decrease in sialylation***											
P00450	Ceruloplasmin^†^										1.8
		EHEGAIYPD**NTT**DFQR^*^	138	2	971.00	1939.99	1939.85	0.14	91	**3.1**	
		AGLQAFFQVQEC**NK**	358	2	864.07	1726.13	1725.79	0.35	99	**0.4**	
		E**NLT**APGSDSAVFFEQGTTR	397	2	1088.42	2174.84	2174.01	0.83	74	**1.9**	
***Proteins with no change in sialylation***											
P02763	Alpha-1-acid glycoprotein 1										1.8
		QDQCIY**NTT**YLNVQR	93	2	982.56	1963.11	1962.90	0.20	78	**1.8**	
P19652	Alpha-1-acid glycoprotein 2										1.0
		QNQCFY**NSS**YLNVQR	93	2	985.07	1968.12	1967.87	0.25	79	**1.0**	
P01011	Alpha-1-antichymotrypsin										1.3
		YTG**NAS**ALFILPDQDK	271	2	927.47	1852.93	1852.89	0.04	73	**1.3**	
P02765	Alpha-2-HS-glycoprotein										1.4
		VCQDCPLLAPL**NDT**R	156	1	1816.60	1815.59	1815.83	-0.24	56	**1.3**	
		KVCQDCPLLAPL**NDT**R	156	2	997.06	1992.10	1991.98	0.12	68	**1.1**	
		AALAAFNAQN**NGS**NFQLEEISR	176	2	1207.45	2412.88	2412.16	0.72	107	**1.6**	
P01008	Antithrombin-III										1.8
		SLTF**NET**YQDISELVYGAK	187	2	1136.45	2270.88	2270.10	0.78	53	**1.8**	
P10909	Clusterin										1.1
		LANLTQGEDQYYLR	374	2	864.74	1727.47	1727.82	-0.35	80	**1.1**	
P00748	Coagulation factor XII										1.4
		**NHS**CEPCQTLAVR	433	2	808.78	1615.55	1615.69	-0.14	69	**1.4**	
P02749	Beta-2-glycoprotein 1										0.8
		VYKPSAG**NNS**LYR	162	2	778.62	1555.22	1554.75	0.47	45	**1.1**	
		LG**NWS**AMPSCK	253	2	680.39	1358.77	1358.60	0.17	56	**1.4**	
P08603	Complement factor H										1.4
		MDGAS**NVT**CINSR	1029	2	745.00	1487.99	1487.63	0.36	49	**1.0**	
		IPCSQPPQIEHGTI**NSS**R	882	2	1033.76	2065.51	2064.97	0.54	90	**1.9**	
P36980	Complement factor H-related protein 2										1.8
		LQNNEN**NIS**CVER	126	2	819.61	1637.20	1636.74	0.46	58	**1.8**	
P08185	Corticosteroid-binding globulin										1.6
		AQLLQGLGF**NLT**ER	96	2	804.87	1607.73	1606.86	0.87	74	**1.6**	
O75636	Ficolin-3										1.1
		VELEDFNGNR	189	2	621.04	1240.06	1239.57	0.50	66	**1.1**	
Q08380	Galectin-3-binding protein										1.5
		ALGFE**NAT**QALGR	69	2	696.82	1391.63	1391.69	-0.06	66	**1.5**	
P05546	Heparin cofactor 2										1.7
		**NLS**MPLLPADFHK	49	2	785.62	1569.22	1568.81	0.41	60	**1.7**	
P19823	Inter-alpha-trypsin inhibitor heavy chain H2										1.0
		GAFIS**NFS**MTVDGK	118	2	781.10	1560.18	1559.70	0.48	93	**1.0**	
P03952	Kallikrein										1.2
		IYSGIL**NLS**DITK	453	2	762.29	1522.57	1522.80	-0.23	90	**1.2**	
P01042	Kininogen-1										1.2
		LNAEN**NAT**FYFK	294	2	740.53	1479.05	1478.70	0.35	80	**1.2**	
		ITYSIVQT**NCS**K	205	2	731.20	1460.38	1460.71	-0.33	64	**1.6**	
		YNSQ**NQS**NNQFVLYR	48	2	967.71	1933.40	1932.88	0.52	0.52	**0.9**	
P05155	Plasma protease C1 inhibitor										1.3
		DTFV**NAS**R	238	2	479.10	956.18	956.45	-0.27	38	**1.3**	
P00734	Prothrombin										1.8
		**NFT**ENDLLVR	416	2	634.62	1267.22	1267.63	-0.41	45	**1.8**	
P04004	Vitronectin										1.2
		**NGS**LFAFR	169	2	485.67	969.32	969.48	-0.16	53	**1.2**	
***Proteins with dicrease in sialylation***											
P0C0L4	Complement C4-A										0.7
		GL**NVT**LSSTGR	1335	2	575.20	1148.39	1148.59	-0.20	72	**1.2**	
		**NTT**CQDLQIEVTVK	1391	2	875.54	1749.07	1748.83	0.24	72	**0.3**	

List of identified glycosylation sites and the results of quantitation of N-deglycosylated peptides obtained from normal and ovarian cancer sera by using LTL quantitative proteomics method.

^*^ The RSD of this method is 9% as determined by the reproducibility studies with fetuin [28].

^†*^ The ratio change in the glycopeptides of these proteins is due to increase in sialylation and not due to the increase in protein concentration.

### Quantitative analysis

**Figure 5:**
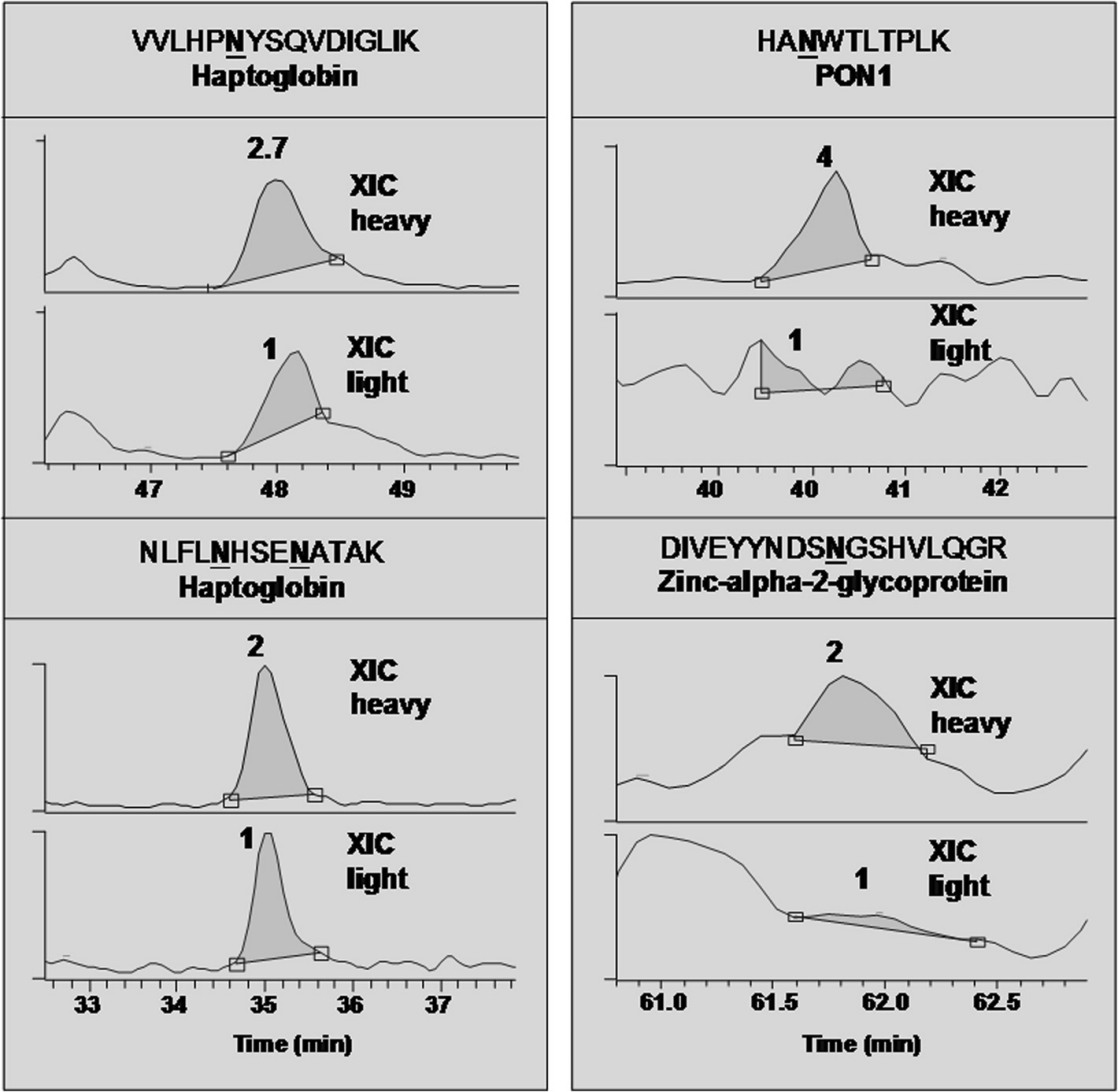
**The XIC’s and quantitative ratios of light and heavy deglycosylated peptides identified in the LTL proteomics analysis of ovarian cancer serum.** These ratios were obtained by calculating the peak areas in the XIC’s of precursor peptides using Xcalibur software.

We investigated the differences in sialylated glycopeptides in normal and ovarian cancer serum samples by quantitating the N-deglycosylated peptides as described in the experimental procedure. Table 1 summarizes the results of the quantification of N-deglycosylated peptides and the corresponding proteins. As evident in Table 1, quantitative ratios are very different and vary significantly between different peptide sequences of each protein suggesting prominent differences at the site of sialylation. For example, the sialylation of glycopeptides VVLHPNYSQVDIGLIK (haptoglobin), NLFLNHSENATAK (haptoglobin), HANWTLTPLK (PON1) and DIVEYYNDSNGSHVLQGR (alpha-2-glycoprotein 1, zinc) are increased by 2.7 fold, 2.0 fold, 4 fold and 2 fold, respectively, as confirmed by the XIC’s of their light and heavy isotopes (Figure 5). Similarly, out of 30 glycoproteins, sialylation is increased in 10 proteins that are identified by multiple N-deglycosylated peptides in ovarian cancer serum samples. However, sialylation is not increased in all the glycopeptides from these up-regulated glycoproteins and. interestingly; we were also able to identify differential sialylation in multiple glycopeptides from a single protein. For example, three N linked glycopeptides (EHEGAIYPDNTTDFQR – 3 fold increase, ENLTAPGSDSAVFFEQGTTR- 1.9 fold increase, AGLQAFFQVQECNK – and 2.2 fold decrease) were identified in ceruloplasmin protein with differential sialylation (Table 1). In contrast, we identified only one glycopeptide, NTTCQDLQIEVTVK with a 3 fold decrease in sialylation and no change was observed in the other glycopeptide, GLNVTLSSTGR, from Complement C4-A protein. Out of 45 glycopeptides identified, sialylation was increased in 10 peptides and decreased in 2 peptides by more than 2 fold in each case. Standard deviation in the current ovarian cancer glycopeptide analysis was assumed to be 9% based on our previous triplicate analysis of Fetuin N-linked glycopeptides using the LTL quantitative proteomics method [28].

In order to determine whether the changes in sialylated glycopeptides are a result of the alterations in the level of sialic acid at the identified site or at the concentration of the parent glycoprotein levels, we performed quantitative analysis of the non-glycosylated peptides obtained from the same samples. These peptides were obtained from the flow- through peptide mixtures of the SNA lectin enrichment step of normal and cancer serum samples. The non-glycosylated peptides were further purified, labeled by iTRAQ reagents (in triplicate), fractionated by SCX chromatography and analyzed by LC-MS/MS experiments (in triplicate) using Orbitrap MS. Quantitative analysis was performed using proteome discoverer software. We identified more than 100 serum proteins out of which 28 glycoproteins were identified in the N-deglycosylated peptide analysis using the LTL method (Table 2). The concentration remained unchanged for all the 28 proteins between normal and ovarian cancer serum samples with overall heavy to light ratios ranged from 0.8 to 1.5. These 28 proteins also include the 9 glycoproteins identified by LTL method in which the sialylation, as analyzed by the glycopeptides, is increased (indicated with asterisk in Table 1). This suggests that sialylation indeed increased in ovarian cancer for the 10 N-linked glycopeptides corresponding to the 9 glycoproteins identified by the LTL method and it is independent of their protein concentration. For the remaining 18 proteins, we observed no change in sialylation or protein concentration levels.

**Table 2:** List of glycoproteins identified and their iTRAQ quantitative ratios obtained from the triplicate analysis of nonglycosylated peptides of normal and ovarian cancer sera

**Swissprot ID**	**Description**	**# Peptides**	**Sequence coverage**	**Protein ratio (Cancer/Normal)**
				**(Mean±Stdev)**
P01011	Alpha-1-antichymotrypsin	6	13.2	1.5±0.03
P01008	Antithrombin-III	7	14.4	1.5±0.07
P02763	Alpha-1-acid glycoprotein 1	4	17.4	1.3±0.14
P04114	Apolipoprotein B-100	63	12.8	1.3±0.09
P00748	Coagulation factor XII	3	4.1	1.3±0.39
P19652	Alpha-1-acid glycoprotein 2	3	12.9	1.3±0.17
P01009	Alpha-1-antitrypsin	12	26.3	1.2±0.23
P00738	Haptoglobin	12	26.4	1.2±0.02
P00450	Ceruloplasmin	11	10.6	1.2±0.02
P25311	Zinc-alpha-2-glycoprotein	5	17.1	1.1±0.02
P05155	Plasma protease C1 inhibitor	3	5.6	1.1±0.03
P36980	Complement factor H-related protein 2	3	11.1	1±0.20
P02790	Hemopexin	7	15.2	1±0.04
P0C0L4	Complement C4-A	26	14.3	1±0.05
P00734	Prothrombin	8	14.8	1±0.04
P08185	Corticosteroid-binding globulin	2	4.0	1±0.10
P02749	Beta-2-glycoprotein 1	2	7.5	0.9±0.08
P27169	Serum paraoxonase/arylesterase 1	5	13.0	0.9±0.05
O75882	Attractin	6	4.3	0.9±0.07
P08603	Complement factor H	17	15.0	0.9±0.11
P10909	Clusterin	6	11.6	0.9±0.03
P19823	Inter-alpha-trypsin inhibitor heavy chain H2	10	9.5	0.8±0.09
P03952	Plasma kallikrein	8	11.9	0.8±0.13
P04004	Vitronectin	4	7.3	0.8±0.03
P05546	Heparin cofactor 2	7	13.2	0.8±0.04
P01042	Kininogen-1	11	16.0	0.8±0.06
P02765	Alpha-2-HS-glycoprotein	7	19.4	0.8±0.03
P02787	Serotransferrin	26	41.4	0.7±0.06

### Western blot analysis

#### Validation of differentially sialylated glycoproteins

**Figure 6:**
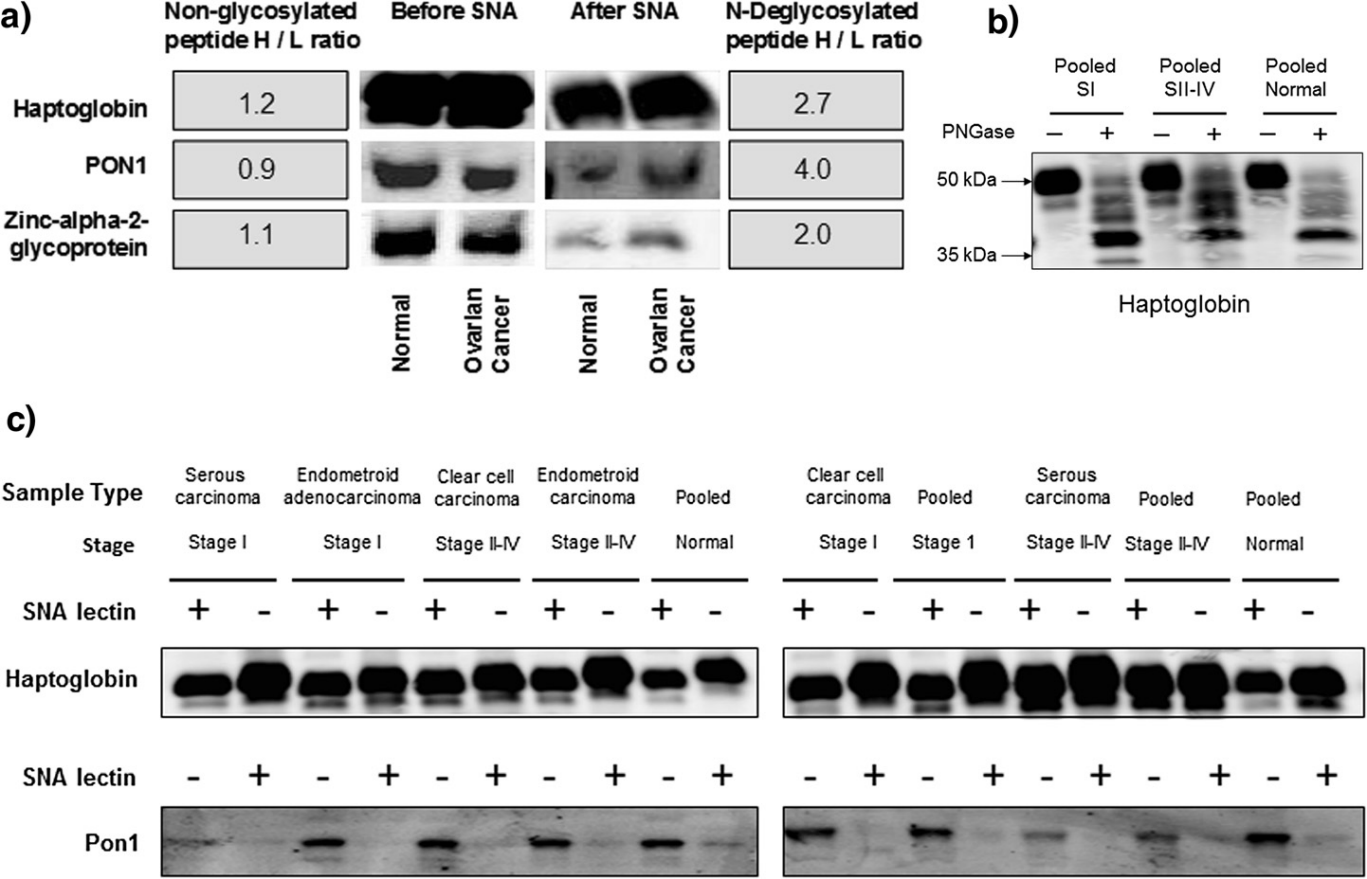
Western blot analysis: a) Validation of differentially sialylated glycoproteins b) Investigation of O-sialylated glycoprotein contribution c) Pooling vs individual patient sample analysis.

To further verify our quantitative results obtained by LTL and iTRAQ methods, western blot analysis was performed using specific antibodies targeting the selected serum glycoproteins. We selected haptoglobin and PON1 [34], based on the implication of their glycosylation, in particular the sialylation aberration in ovarian cancer and other cancers. Zinc-alpha-2-glycoprotein was chosen due to its significant biomarker potential for various cancer indications [35]. Western blot analysis was performed using proteins obtained before and after the lectin enrichment of normal and cancer serum samples (Figure 6). The data indicates that before SNA enrichment, there is no significant difference in the levels of haptoglobin and zinc-alpha-2-glycoprotein between the normal and cancer serum samples, whereas cancer serum contained lower levels of the PON1 glycoprotein. The western data is in agreement with the non-glycosylated peptide analysis, which showed no difference between normal and cancer samples at the protein level. However, after SNA lectin enrichment, haptoglobin, PON1 and zinc-alpha-2-glycoproteins show a significant increase in protein concentration and their levels are comparable to the deglycosylated peptide H/L ratios (Figure 6a) indicating that the sialylation is indeed increased in these proteins in ovarian cancer. Overall, the western blot data corroborates the LTL quantitative ratios of the deglycosylated peptides. The minor differences between the western blot data and the glycopeptide quantitation results may be attributed to the contribution of the O-linked sialylated fraction of each protein which is further investigated as described below.

#### Individual patient sample analysis

In order to ascertain the effect of pooling of the patient samples on the sialylation differences between normal and cancer, we have performed western blot analysis of individual samples obtained from serous, endometroid and clear cell carcinoma of stage I and Stage II-IV ovarian cancer patients. These samples were subjected to SNA lectin enrichment and probed with PON1 and haptoglobin antibodies. We also ran the pooled stage I and stage II-IV samples and pooled healthy female samples as controls and the corresponding data is given in Figure 6b. We observed no significant differences between the stages and types of ovarian cancers. In general, there was only a small difference between the cancer samples and the healthy controls at the glycoprotein level as assessed by the western blot.

#### Investigation of O-sialylated glycoprotein contribution

We have also carried out PNGase (+/−) experiments after SNA enrichment of intact glycoproteins and western blot analysis using haptoglobin specific antibodies. As illustrated in Figure 6c, multiple bands were identified in the PNGase (+) experiment from pooled normal and cancer (SI and SII-IV) samples. The identification of multiple bands may be explained due to the glycosylation heterogeneity in haptoglobin and partial cleavage of N-linked glycans by PNGase treatment. However, only a very low intense band was identified at the 50 kDa region in PNGase (+) experiment as compared to the PNGase (−) experiment. This observation is true for both the normal and cancer (SI and SII-IV) samples, although the intensity of the 50 kDa band in PNGase (+) experiment is slightly higher in case of the cancer sample. This suggests that the amount of O-linked sialylated haptoglobin contributing to the N-linked sialylated haptoglobin in SNA enrichment process is very low and it may not have a major impact in the determination of sialylation differences at the glycopeptides level between normal and cancer. Thus we have proved that the sialylation differences in N-linked glycopeptides of haptoglobin are indeed true and significant.

## Discussion

The strategy involving the investigation of glycan specific changes at the glycopeptide level has many advantages and it is indeed important over examining the same changes at the protein level. It is very well established that the glycosylation changes are unique to a particular site in a glycoprotein and its association with various cancers. It is not possible to accurately examine these changes at the glycoprotein level due to the high glycoprotein heterogeneity and technical limitations. Enrichment of intact glycoproteins using lectins may be comprised by a mixture of N-linked and O-linked protein fractions in different ratios. This leads to ambiguity in the assessment of their actual concentrations by validation experiments like western blot. Therefore, prior investigation of the dynamic glycosylation changes at the glycopeptide level is imperative to discover potential cancer specific biomarkers.

Lectin enrichment has been widely used to augment glycoprotein biomarker discovery in various cancers [7,27,28]. In order to investigate sialic acid aberrations, SNA lectin was used to enrich sialic acid containing glycoproteins in ovarian cancer. Glycosylation differences in N-linked glycopeptides was analysed by finding differences indirectly in deglycosylated peptides without compromising the glycan selection. Recently Ueda et al. [27], developed a novel isotopic glycosidase elution and labeling on lectin-column chromatography (IGEL) method, which is equally useful to investigate glycosylation changes at the peptide level and up to 8 samples can be analyzed simultaneously using iTRAQ technology. Herein, we used the LTL method to investigate the global sialylation changes in ovarian cancer on each glycoprotein via deglycosylated peptide analysis. There are many advantages associated with the LTL method [28] in probing the glycosylation changes at the glycopeptide level. In this study, we observed the consensus NXS/T sequence and an ^18^O tag in each glycopeptide identified, which significantly increased the confidence and enabled the identification of true positive N-glycopeptides based on the 3Da mass shift at the glycosylation site. This approach not only simplified the complexity of various glycopeptides, but also increased the sensitivity considerably to identify a particular sialylated peptide with high quantitative accuracy on the sialylation changes.

Quantitative analysis of deglycosylated and non-glycosylated peptides revealed a significant increase in sialylation of the glycoproteins while the concentration of the glycoproteins remained unchanged in ovarian cancer (Table 1). Western blot analysis of SNA enriched selected glycoproteins further confirmed this observation (Figure 6). Our results are further supported by reports in the literature on the implication of the selected proteins in various cancers including ovarian cancer. Up-regulation of glycosylation in haptoglobin has been reported in pancreatic cancer [36-38]. Glycopeptide, NLFLNHSENATAK from haptoglobin has been reported to have de-sialylated, mono-sialylated and completely sialylated glycan structures, while, only the completely sialylated structure has been shown to be up-regulated in pancreatic cancer samples compared to the controls [7]. In addition to the changes in glycosylation, elevation of the haptoglobin protein levels in the serum and ascitic fluid of ovarian cancer patients has been reported in previous studies [38,39]. Similarly, PON1 has been implicated in ovarian cancer and it was shown to be involved in a variety of enzymatic activities including lactonase, arylesterase, peroxidase, and phospholipase activities [34]. It has been suggested that women who carry PON1 alleles associated with reduced PON1 activity and those with lower concentrations might be at higher risk for developing ovarian cancer [40]. It was also reported that PON1 genetic variants may alter the risk of epithelial ovarian cancer among smokers and obese women. Zinc-alpha-2-glycoprotein has been proposed as one of the biomarkers for predicting the response to thalidomide-based therapy in newly diagnosed multiple myeloma patients [41]. It is also a potential molecular marker for biochemical relapse in men with margin-positive, localized prostate cancer [42], and differential regulation of this protein has been linked to gastrointestinal cancer [43], prostate cancer [44], cancer cachexia [45], and bladder cancer [46]. However, no glycosylation aberration was reported for this protein and linked to any cancer including ovarian cancer. On the contrary, we found significant increase in sialylation in our deglycosylated peptide and western blot analysis of SNA enriched ovarian cancer serum samples. Our data suggests that glycosylation aberrations, as assessed at the glycopeptides level, are a highly sensitive and specific tool for biomarker discovery in various cancers.

Significant sialylation aberrations were observed in ovarian cancer when we compared our quantitative results on the sialylated glycopeptides corresponding to the glycoproteins reported in the literature. Similar to our findings, changes in glycosylation have been reported for alpha-1-antitrypsin glycoprotein in ovarian cancer patients [47]. Glycosylation changes in serotransferrin have not been reported earlier in ovarian cancer. However, ovarian cancer cells have been shown to secrete serotransferrin in chemically defined medium and up-regulation of serum levels of four isoforms of serotransferrin have been observed in 42% of cancer patients after six cycles of chemotherapy [48]. In Our study in which significant changes in serotransferrin sialylation were observed in multiple glycopeptides in ovarian cancer (Table 1), along with published reports suggesting serotransferrin could be a potential biomarker for ovarian cancer at both glycoprotein and glycopeptides level. Furthermore, Liu et al. [49], reported significant sialylation changes in TLNQSSDELQLLSMGNAMFVK glycopeptide and no change in the LSLGAHNTTLTEILK glycopeptide, from alpha-1-antichymotryspin glycoprotein in ovarian cancer. In our analysis, we identified a different sialylated peptide, YTG**N**ASALFILPDQDK from alpha-1-antichymotryspin with no change in silaylation.

Evaluation of individual vs pooled samples of various types and stages of ovarian cancer by western blot analysis revealed no differences at the glycoprotein level suggesting there is minimal loss of significance in using pooled samples for discovery of biomarkers. However, extensive validations have to be performed with significant number of individual samples prior to the selection of potential biomarkers for diagnostics development. In general, our data is in accordance with previously reported studies and emphasizes that the sialylation status of the glycoproteome in cancer is highly dependent on the type of samples compared and disease status and is independent of the concentration of the parent glycoproteins.

**Figure 7:**
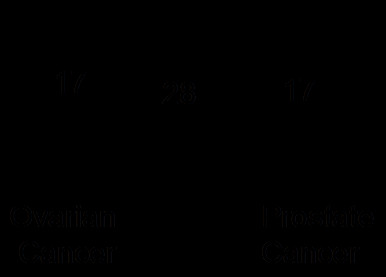
Venn diagram showing the overlap and unique desialylated peptides identified in ovarian cancer sera and prostate cancer sera.

We recently reported the sialylation aberrations in prostate cancer serum samples by employing the LTL method and unambiguously identified and quantitated 45 N-linked sialylated glycopeptides. In this study, we compared the N-linked sialylated glycopeptidome of prostate cancer serum samples with ovarian cancer data. The results of the comparative analysis of all the glycopeptides identified in these two cancers are given in Table 3. Interestingly, a majority of the glycopeptides identified in prostate cancer was also identified in ovarian cancer; however, we observed significant differences in the sialylation of most of these glycopeptides in these two cancers. While the sialylation of 6 glycopeptides increased in both cancers, the sialylation of 5 other glycopeptides increased only in ovarian cancer. In addition, the sialylation is significantly elevated in 16 glycopeptides only in prostate cancer suggesting the differential prevalence of sialylation aberration in various cancers. Although 28 glycopeptides were common to these two cancers, 17 glycopeptides were uniquely identified in ovarian cancer and 17 different glycopeptides were uniquely identified in prostate cancer (Figure 7). The data is intriguing as to the potential of glycosylation aberrations at the glycopeptide level as cancer specific biomarkers.

**Table 3:** List of differentially sialylated glycopeptides identified and quantitated in ovarian cancer and prostate cancer sera

**Swissprot ID**	**Protein**	**Glycopeptide sequence**	**Heavy/Light ratio (peptide)**	
			**Ovarian cancer**	**Prostate cancer**
***increase of sialylation in ovarian cancer and prostate cancer***				
P00738	Haptoglobin	NLFL**NHS**E**NAT**AK	**2.0**	**3.1**
		VVLHP**NYS**QVDIGLIK	**2.7**	**3.8**
P00450	Ceruloplasmin	EHEGAIYPD**NTT**DFQR	**3.1**	**3.9**
P02790	Hemopexin	ALPQPQ**NVT**SLLGCTH	**2.1**	**3.8**
P04114	Apolipoprotein B-100	FVEGSH**NST**VSLTTK	2.0	2.9
P01009	Isoform 1 of Alpha-1-antitrypsin	YLG**NAT**AIFFLPDEGK	**2.1**	**5.5**
***increase of sialylation only in ovarian cancer***				
P27169	Serum paraoxonase/arylesterase 1	HA**NWT**LTPLK	**4.0**	**▬**
P02787	Serotransferrin	CGLVPVLAENY**NK**	**7.6**	**1.1**
		QQQHLFGS**NVT**DCSGNFCLFR	**3.1**	**▬**
O75882	Attractin	**NHS**CSEGQISIFR	**2.2**	**▬**
P25311	Zinc-alpha-2-glycoprotein	DIVEYYNDSNGSHVLQGR	**2.0**	**▬**
***increase of sialylation only in prostate cancer***				
P36980	Complement factor H-related protein 1	LQNNEN**NIS**CVER	**1.8**	**2.2**
P19823	Inter-alpha-trypsin inhibitor heavy chain H2	GAFIS**NFS**MTVDGK	**1.0**	**4.2**
P08603	Isoform 1 of Complement factor H	IPCSQPPQIEHGTI**NSS**R	**1.9**	**3.5**
		ISEENETTCYMGK	**▬**	**2.8**
		MDGAS**NVT**CINSR	**1.0**	**2.3**
P04004	Vitronectin	NNATVHEQVGGPSLTSDLQAQSK	**▬**	**2.4**
P02765	Alpha-2-HS-glycoprotein	AALAAFNAQN**NGS**NFQLEEISR	**1.6**	**3.7**
		KVCQDCPLLAPL**NDT**R	**1.1**	**4.3**
		VCQDCPLLAPL**NDT**R	**1.3**	**2.4**
P02763	Alpha-1-acid glycoprotein 1	QDQCIY**NTT**YLNVQR	**1.8**	**2.1**
P00450	Ceruloplasmin	E**NLT**APGSDSAVFFEQGTTR	**1.9**	**3.1**
		AGLQAFFQVQEC**NK**	**0.4**	**6.8**
P02790	Hemopexin	SWPAVG**NCS**SALR	**1.6**	**4.4**
P27169	Serum paraoxonase/arylesterase 1	VTQVYAE**NGT**VLQGSTVASVYK	**▬**	**2.1**
P01042	Isoform HMW of Kininogen-1	ITYSIVQT**NCS**K	**1.6**	**2.5**
		LNAEN**NAT**FYFK	**1.2**	**5.0**
P01871	IGHM protein	GLTFQQ**NAS**SMCVPDQDTAIR	**▬**	**4.7**
		YK**NNS**DISSTR	**▬**	**2.3**
***increase of sialylation in ovarian cancer and prostate cancer***				
P00738	Haptoglobin	NLFL**NHS**E**NAT**AK	**2.0**	**3.1**
		VVLHP**NYS**QVDIGLIK	**2.7**	**3.8**
P00450	Ceruloplasmin	EHEGAIYPD**NTT**DFQR	**3.1**	**3.9**
P02790	Hemopexin	ALPQPQ**NVT**SLLGCTH	**2.1**	**3.8**
P04114	Apolipoprotein B-100	FVEGSH**NST**VSLTTK	**2.0**	**2.9**
P01009	Isoform 1 of Alpha-1-antitrypsin	YLG**NAT**AIFFLPDEGK	**2.1**	**5.5**
***increase of sialylation only in ovarian cancer***				
P27169	Serum paraoxonase/arylesterase 1	HA**NWT**LTPLK	**4.0**	**▬**
P02787	Serotransferrin	CGLVPVLAENY**NK**	**7.6**	**1.1**
		QQQHLFGS**NVT**DCSGNFCLFR	**3.1**	**▬**
O75882	Attractin	**NHS**CSEGQISIFR	**2.2**	**▬**
P25311	Zinc-alpha-2-glycoprotein	DIVEYY**NDS**NGSHVLQGR	**2.0**	**▬**
***increase of sialylation only in prostate cancer***				
P36980	Complement factor H-related protein 1	LQNNEN**NIS**CVER	**1.8**	**2.2**
P19823	Inter-alpha-trypsin inhibitor heavy chain H2	GAFIS**NFS**MTVDGK	**1.0**	**4.2**
P08603	Isoform 1 of Complement factor H	IPCSQPPQIEHGTI**NSS**R	**1.9**	**3.5**
		ISEE**NET**TCYMGK	**▬**	**2.8**
		MDGAS**NVT**CINSR	**1.0**	**2.3**
P04004	Vitronectin	N**NAT**VHEQVGGPSLTSDLQAQSK	**▬**	**2.4**
P02765	Alpha-2-HS-glycoprotein	AALAAFNAQN**NGS**NFQLEEISR	**1.6**	**3.7**
		KVCQDCPLLAPL**NDT**R	**1.1**	**4.3**
		VCQDCPLLAPL**NDT**R	**1.3**	**2.4**
P02763	Alpha-1-acid glycoprotein 1	QDQCIY**NTT**YLNVQR	**1.8**	**2.1**
P00450	Ceruloplasmin	E**NLT**APGSDSAVFFEQGTTR	**1.9**	**3.1**
		AGLQAFFQVQEC**NK**	**0.4**	**6.8**
P02790	Hemopexin	SWPAVG**NCS**SALR	**1.6**	**4.4**
P27169	Serum paraoxonase/arylesterase 1	VTQVYAE**NGT**VLQGSTVASVYK	**▬**	**2.1**
P01042	Isoform HMW of Kininogen-1	ITYSIVQT**NCS**K	**1.6**	**2.5**
		LNAEN**NAT**FYFK	**1.2**	**5.0**
P01871	IGHM protein	GLTFQQ**NAS**SMCVPDQDTAIR	**▬**	**4.7**
		YK**NNS**DISSTR	**▬**	**2.3**

List of differentially sialylated glycopeptides identified and quantitated in ovarian cancer and prostate cancer sera by using LTL quantitative proteomics method.

## Conclusions

We successfully used LTL and iTRAQ quantitative proteomics methods in conjunction with western blot analysis to investigate the sialylation aberration in N-linked glycopeptides in normal and ovarian cancer serum samples. In the current study, we identified 30 glycoproteins with 45 N-linked sialylated glycopeptides comprising 46 glycosylation sites. We observed increases in sialyation of 10 N-linked glycopeptides in ovarian cancer, which is independent of their protein concentrations as confirmed by the non-glycosylated peptide analysis. Sialylation change in some of the selected peptides was also confirmed by the western blot analysis of SNA enriched precursor glycoproteins. Overall, we observed a good correlation between the proteomic analysis and western blot experiments. These integrated results strongly suggest that haptoglobin, PON1 and zinc-alpha-2-glycoprotein may undergo specific sialylation changes at the glycoeptide level in ovarian cancer.

Further, the comparative analysis of N-linked sialylated glycopeptides from ovarian and prostate cancer serum samples revealed major variation in the level of sialylation in these two cancers. The increased cancer specific sialylation in the N-glycopeptides in these two cancers also implies that the glycopeptides are potential targets for biomarker discovery. These preliminary observations were further confirmed by screening individual samples of various types and stages of ovarian cancer by western blot analysis, and the current data clearly demonstrate that the sialylation changes are unique to each N-linked glycopeptide glycosylation in a particular type of cancer and this approach may have a high potential for the development of early diagnostic strategies for cancer in general.

## Methods

### Serum samples

Serum samples were procured from ten patients with histologically confirmed ovarian carcinoma (primary diagnosis, regardless of stage of the disease), prior to surgery and chemotherapy. Patient samples were obtained under IRB approved protocols from patients undergoing treatment at Duke University Medical Center. Equal number (4 each) of serous adenocarcinoma, endometroid adenocarcinoma and clear cell carcinoma ovarian cancer samples were used. Serum samples from five age matched healthy female control individuals were purchased from Research Blood Components, LLC (Brighton, MA). Equal amounts of undiluted serum from each cohort were used to generate composites consisting of cancer and normal serum samples for the study.

### Chemicals and reagents

HPLC grade acetonitrile (ACN) and water were obtained from Burdick and Jackson (Muskegon, MI). Methanol, calcium chloride (CaCl_2_) and sodium phosphate (NaH_2_PO_4_) were purchased from EMD chemical Inc. (Gibbstown, NJ). Ethanol was purchased from Decon Labs, Inc. (King of Prussia, PA). Acetic anhydride (^1^H_6_/^2^D_6_), H_2_^18^O water, phenylmethanesulfonyl fluoride (PMSF), ammonium bicarbonate (AB), formic acid (FA), trifluoroacetic acid (TFA), dithiothreitol (DTT), iodoacetamide (IA), manganese chloride (MnCl_2_), lactose were purchased from Sigma (St. Louis, MO). *Sambucus nigra* lectin (SNA) agarose was purchased from Vector Laboratories (Burlingame, CA). Spin-x columns were purchased from Corning, Inc. (Lowell, MA). Peptide-*N*-glycosidase F (PNGase F) was purchased from QA BIO (Palm Desert, CA). Protein A/G beads and C-18 miniprep columns were purchased from Thermo Scientific (Waltham, MA). RapiGest SF, vacuum manifold and C-18 sep-pak cartridges were obtained from Waters (Milford, MA). Tris, NaCl and NaOH were purchased from Fisher (Pittsburgh, PA). PBS was purchased from Mediatech, Inc. (Manassas, VA). Ultra Centricon 3 kDa filters were purchased from Millipore (Billerica, MA). Trypsin was purchased from Promega (Madison, WI). iTRAQ labeling kit was purchased from Applied Biosystems (Bedford, MA).

### In-solution digestion

Prior to the in-solution digestion, serum samples were pre-cleared with spin-x columns and IgG was removed using protein A/G beads and buffer exchanged with 50 mM AB using Ultra Centricon 3 kDa filters. The protein concentrations of normal and cancer serum samples were calculated by nanodrop technologies instrument (Wilmington, DE).

Briefly, the protein solution was mixed with 50 μl of 50 mM AB containing 0.1% of Rapigest SF and the protein was reduced with DTT (5 μm/μl in 50 mM AB) by incubating the mixture at 65°C for 45 min and alkylated with IA (15 μm in 50 mM AB) by incubating the reaction mixture in dark for 30 min. Then the alkylated glycoproteins were digested by trypsin (5 ng/μl in 50 mM AB) overnight at 37°C in a water bath. RapiGest was removed according to the vendor’s recommended protocol.

### Enrichment of glycopeptides using SNA lectin

Tryptic peptides were mixed with 1 mM solution of PMSF prior to the glycoprotein enrichment by SNA lectin. The tryptic peptide sample was diluted to 4:1 with 5 × binding buffer (100 mM Tris, 750 mM NaCl, 5 mM CaCl_2_ and 5 mM MnCl_2_, pH 6.7). The lectin columns were prepared by adding 200μl of SNA lectin to the cartridges and spun down at 1000 × g for 1 min to remove storage buffer. The lectin resin in columns was washed with 200 μl (2 ×) of 1 × washing buffer (20 mM Tris, 150 mM NaCl, 1mM CalCl_2_ and 1 mM MnCl_2_). Then the sample was loaded onto the columns, mixed and incubated at room temperature (RT) for 10 min. The columns were centrifuged to collect the flow through and washed with 400 μl of 1 × washing buffer (2 ×). Again, 400 μl of 1 × washing buffer was added and incubated for 5 min at RT (2 ×). The flow through from samples was saved for additional labeling experiments. Finally, the glycopeptides were eluted from the lectin columns by adding 200 μl (2 ×) of elution buffer (20 mM Tris, 150 mM NaCl, 1mM CalCl_2_, 1 mM MnCl_2_ and 200 mM lactose) and incubating for 10 min at RT. The flow through containing the glycopeptides were collected by centrifugation and purified by C-18 chromatography.

### Acetylation of glycopeptides

Glycopeptides obtained from normal and ovarian cancer serum samples and non-glycosylated serum peptides (lectin column flow through) were labeled with acetyl (^1^H_3_/^2^D_3_) groups according to a published protocol [50]. Briefly, 2 μl of ^1^H_6_-acetic anhydride or ^2^D_6_-acetic anhydride [50% (vol/vol) in methanol] were added to 100 μl of peptide mixture in 50% methanol/water (vol/vol). The mixture was allowed to react at 22°C for 15 min. The reaction was stopped by the addition of 2.2 μl of formic acid and equal aliquots of both samples were mixed.

### PNGase F digestion of glycopeptides

The light (normal) and heavy (cancer) N-terminus/lysine labeled serum glycopeptides were mixed and digested with PNGase F to cleave the N-glycans from peptides according to the protocol described elsewhere [51]. Briefly, the glycopeptides were dried in vacuum and re-dissolved in 100 mM NaH_2_PO_4_ buffer (pH 7.5). The PNGase F enzyme was dissolved in 50 mM AB. NaH_2_PO_4_ buffer solution and AB buffer solution were prepared in H_2_^18^O before dissolving glycopeptides and enzyme, respectively. The reaction mixture was incubated with 6 μl of PNGase F at 37°C overnight. The reaction was stopped by adding 0.5% TFA solution. The resulting N-deglycosylated peptides were purified by C-18 chromatography.

### Purification of peptides by C-18 chromatography

The trypsin digested peptides, SNA enriched glycopeptides and N-deglycosylated peptides were purified by C-18 reversed-phase (RP) chromatography using C-18 spin columns with the help of a vacuum manifold. Briefly, the C-18 columns were activated with 50% acetonitrile and equilibrated with a buffer containing 2% ACN and 0.1% TFA in water. Then peptide mixture (dissolved in 2% ACN and 0.1% TFA in water) was slowly loaded into the C-18 cartridge. Then the columns were washed thoroughly with 0.1% TFA to remove salts and buffers. Finally, the tryptic peptides were eluted with 70% acetonitrile twice (250 μl each), the two eluted fractions were combined and concentrated. The serum glycopeptide mixture was fractionated by C-18 RP column (4.6 mm diameter × 150 mm length) using an offline ultimate 3000 HPLC (Dionex, Sunnyvale, CA). Mobile phase A was 2% acetonitrile (ACN) and 0.1% formic acid (FA) in water, while mobile phase B was 0.1% FA and 90% ACN in water. Peptides were then eluted from the column with an 80 min linear gradient from 5 to 80% buffer B at a flow rate of 200μL/min. A total of 35 fractions were collected and each fraction was concentrated to 6 μl under vacuum.

### iTRAQ labeling of non-glycosylated peptides

Peptide mixtures were labeled using iTRAQ reagents following the vendors recommended protocol. Briefly, each vial of iTRAQ Reagent was allowed to reach room temperature and spun to bring the solution to the bottom of the tube. 70 μL of ethanol was added to each iTRAQ reagent vial. Then the solution in each vial was vortexed to mix and then spun. The contents of one iTRAQ reagent vial were transferred to one sample tube. Each tube containing the reaction mixture was vortexed to mix and then spun. Finally, the reaction mixtures were incubated at room temperature for 1 hour and then combined and processed further.

### Purification and fractionation of iTRAQ labeled non-glycosylated peptides by SCX chromatography

The iTRAQ labeled peptides mixture was purified and fractionated by an off-line HPLC using SCX analytical column (PolySULFOETHYL A, 200×4.6mm; 5um, 300°A) (Poly LC Inc.). First, the concentrations of buffer salts and organics in iTRAQ labeled peptide solution were reduced by diluting the sample mixture by 10 fold with cation exchange buffer-A (10 mM potassium phosphate (KH_2_PO_4_) in 25% acetonitrile at pH 3.0). The sample mixture was reconstituted with 1 mL cation exchange buffer-load and vortexed to mix. An aliquot was removed and the pH was adjusted by adding 1M HCl to bring it down to pH ~3. To remove the impurities from the peptide mixture, the gradient kept at 5% B in the first 20min and did not collect any fractions. Then the peptides were eluted by buffer–B (10 mM KH_2_PO_4_ in 25% acetonitrile/350 mM potassium chloride (KCl) at pH 3.0) with the gradient of 0% to 95% B in 70 min and 95% to 95% B from 70 min to 80 min at a flow rate of 200μL/min.

### Mass spectrometry analysis

A 3000 nano ultimate HPLC (Dionex, Sunnyvale, CA) was coupled with LTQ mass spectrometer (Thermo Electron, San Jose, CA) equipped with advanced nanospray source to analyze acetyl (^1^H_3_/^2^D_3_) and ^18^O labeled peptides. The serum N-deglycosylated peptide fractions were injected into LC-MS/MS system to identify deglycosylated peptides. As a part of on-line sample clean-up step, the peptides were first concentrated in a C-18 RP trap column (Dionex) and then separated by using a 75μm ID × 15cm C-18 RP analytical column (Dionex, Sunnyvale, CA) equilibrated in 4% ACN/0.1% FA at 250 nL/min flow rate. Mobile phase A was 2% ACN and 0.1% FA in water, while mobile phase B was 0.1% FA and 90% ACN in water. Peptides were separated with a 4% to 50% B in 60 min and 50% to 80% in 90 min and eluted directly into an LTQ MS. The mass range in MS mode was 350 Da to 1800 Da and in MS/MS mode it was set as 100 Da to 2000 Da. N-deglycosylated peptides were analyzed by normal data dependent mode method in which the instrument was set to acquire fragment ion (MS/MS) spectra on the 4 most abundant precursor ions from each MS scan with a repeat count set of 1 and duration of 30 sec. Dynamic exclusion was enabled for 180 sec and the collision energy was 33. In a separate experiment, serum non-glycosylated peptide mixtures were analyzed by an LC-MS/MS experiment (in triplicate) with a long HPLC linear gradient (4% to 50% B in 120 min and 50% to 80% in 180 min).

The iTRAQ labeled non-glycosylated peptides were analyzed by data dependent nano LC-MS/MS experiments on an Velos LTQ-Orbtrap mass spectrometer (Thermo Fisher) interfaced with a nano ultimate HPLC (Dionex). The sample was loaded onto a trap column of 100 um ID X 2 cm (L) packed with 5-μm Magic C18 AQ (200 A, 3 um, Michrom) and washed using 98% H_2_O, 2% ACN, 0.05% TFA buffer at a flow rate of 10 ul/min for 5 min. The peptides were then separated by a self-packed 75um ID X 50 cm (L) fused silica column packed with 3-μm Magic C18 AQ (200 A, 3 um, Michrom) using a linear gradient of Buffer B from 4% to 55% in 50 min at a flow rate of 300 nl/min. The buffer compositions were as follows: Buffer A was 0.1% formic acid in water, buffer B was 0.1% formic acid, 80% acetonitrile. The analytical column was coupled to the mass spectrometer via a nano ion source (Proxeon) with a metal emitter. The peptides were analyzed in the Orbitrap operated at 60,000 resolution in full scan (300–2000m/z) followed by 10 Data-Dependent CID MS/MS scans (100–2000m/z) with 7,500 resolution and normalized collision energy of 45%. Survey scans were acquired in profile mode and MS/MS scans were acquired in centroid mode. Maximum injection times for MS and MS/MS were set to 500 and 1000ms, respectively. The precursor isolation width was set at ±1.2 Da and monoisotopic precursor selection was enabled to exclude singly charged ions from MS/MS. The minimum intensity threshold for MS/MS fragmentation in the Orbitrap analyzer was 5000 counts and the dynamic exclusion was set to 60 sec with repeat count as one.

### Glycopeptide identification and quantitation

The raw data were converted into DTA files using Bioworks 3.1 software (Thermo electron, San Jose, CA). All the ‘DTA’ files were merged and converted into a single text file using an in-house software program. The proteins were identified by searching this text file of tandem mass spectrometry data in Swissprot human database with mascot 2.0 software (MatrixScience, London, U.K). The database search parameters were: mass type-monoisotopic precursor and fragment, enzyme–trypsin, threshold–100, peptide tolerance–1.5 Da, and fragment ion tolerance–1.0. Variable modifications: N-terminus–acetyl (^1^H_3_/^2^D_3_), C–carbamidomethylation; K–acetyl (^1^H_3_/^2^D_3_), N-deamidation (3 Da), M-oxidation, N-PNGase^18^O. The search results, as well as the raw tandem mass spectrometry data were further verified manually and all glycopeptide sequences were identified with high confidence based on the presence of NXT/S motif and a 3 Da mass shift in b or y ions due to the modification at the N-terminus or K, respectively, by heavy acetyl (^2^D_3_) group.

The isotope ratios of light and heavy forms of glycopeptides were calculated manually by Xcalibur software (Thermo Electron, San Jose, USA) using peak areas derived from the XIC’s and dividing the area of the heavy isotope by the area of the light. Similarly, acetyl (^1^H_3_/^2^D_3_) and ^18^O labeled glycopeptides in the serum analysis were quantified manually by using Xcalibur software program In case of multi-peptide protein identification, quantification was performed for at least one glycopeptide of each modification (acetyl (^1^H_3_/^2^D_3_)+^18^O/acetyl (^1^H_3_/^2^D_3_)+^18^O+M-oxdation/acetyl (^1^H_3_/^2^D_3_)+^18^O+esterification). The ratios of the light and heavy isotope-labeled peptides were determined by calculating the peak areas found in the corresponding extracted ion chromatograms (XIC’s) of deglycosylated peptides obtained with 1 Da mass window. These peptide ratios were also confirmed by the relative intensities of multiply charged parent peptide peaks in the MS spectrum. Mean ratios were also calculated for the peptides identified with multiple modifications as light and/or heavy forms. Final protein ratios were calculated by averaging the peptide ratios of all modifications. Since there is only a 3 Da mass difference between differentially labeled deglycosylated peptides and overlapping of light and heavy isotopic envelops could introduce small errors in the quantitation, we considered sialylation is increased in a peptide only if heavy/light ratio of that peptide is ≥ 2 fold.

### Protein identification and quantitation based on the non-glycosylated peptide analysis

Glycopeptides and their corresponding proteins were identified by searching the LC-MS/MS raw data using proteome discoverer software (v 1.3) with Sequest search algorithm (Thermo). The data generation and database search parameters were: m.wt range: 375 Da-1500 Da, threshold: 200 counts, charge state—auto, MS^n^ level—MS^2^, activation type—HCD, precursor ion tolerance: 20 ppm, fragment ion tolerance – 0.02 Da, minimum ion count: 10, in databse - Swissprot human, oxidation of M: 16 Da, N-terminus/K modification by iTRAQ reagents: 144 Da, enzyme - trypsin. The search results were also verified manually to confidently identify the correct peptide sequence. Quantitation was done by selecting a 4-plex iTRAQ default method which contains the isobaric correction parameters for all the iTRAQ labels, however, only 3 labels (114,115,116) were selected for quantitation of non-glycosylated peptides. Peptides quantified with ≥ 1.5 fold increase and with ≤ 1.5 fold decrease in concentration were considered as up regulated and down regulated, respectively, in the current analysis.

### Western blot analysis

#### Validation of differentially sialylated glycoproteins

For each selected protein, 10 ug of each of the indicated serum samples were resolved on a 10% Tris-Glycine gel and the proteins were transferred onto PVDF membrane using a wet transfer apparatus. Various proteins in the serum (Haptoglobin, PON1, Zinc Alpha 2 glycoprotein) were probed with a 1:1000 dilution of commercial antibodies in Odyssey blocking buffer + 0.1% Tween 20 [Sigma, catalog #H8636 (anti-Haptoglobin); Abcam, catalog #ab24261 (anti-PON1); Abcam, catalog #ab47116 (anti-zinc alpha 2 glycoprotein)] followed by a 1:5000 dilution of secondary reagents in Odyssey blocking buffer + 0.1% Tween 20 [Licor Biosciences, catalog #926-32210 (anti-mouse IgG IRDye800CW) or catalog #926-32211 (anti-rabbit IgG IRDye 800CW)]. Blot was developed using fluorescence detection on the Odyssey Infrared Imaging System from Licor Biosciences.

#### Individual patient sample analysis

Western blot analysis was also performed on individual samples obtained from serous, endometroid and clear cell carcinoma of stage I and Stage II-IV ovarian cancer patients. These samples were subjected to SNA lectin enrichment and probed with PON1 or haptoglobin antibodies. Pooled stage I and stage II-IV samples were also subjected to western blot experiments where pooled healthy female sample used as control. Serum samples were analyzed individually or pooled (Before) and 100uL of these were enriched using SNA Lectin as per the product protocol (Vector Labs AL-1303) (After). Following enrichment, approximately 300ug were taken and treated with PNGase in the presence of a 1× protease inhibitor cocktail and 5% NP40 for 2 hours at 37°C. Following treatment with SNA, the protein concentrations were determined using the BCA Assay. Samples were loaded on a 10% TrisGlycine gel as follows - for PON1, 5ug of the Before samples and 2.4ug of the After samples were loaded/well; for Haptoglobin, 1ug of each sample was loaded/well, and the proteins were transferred onto PVDF membrane using a wet transfer apparatus. The membranes were blocked using Odyssey Blocking Buffer and probed with Haptoglobin (Sigma H8636) and PON1 (abcam ab24261) antibodies, followed by a corresponding IRDye secondary antibody (Licor Biosciences #926-32210 or #926-68021). The blot was developed using the Odyssey Infrared Imaging System (Licor Biosciences).

#### Investigation of O-sialylated glycoprotein contribution

Glycoproteins were enriched from 100uL each of the pooled serum samples using SNA Lectin as per the product protocol (Vector Labs AL-1303). Following enrichment, approximately 300ug of the sample was treated with PNGase in the presence of a 1× protease inhibitor cocktail and 5% NP40 for 2 hours at 37°C. After treatment with SNA and/or PNGase, the protein concentrations were determined using the BCA Assay. Ten microgram of the sample was loaded/well on a 10% TrisGlycine gel and the proteins were transferred onto PVDF membrane using a wet transfer apparatus. The membranes were blocked using Odyssey Blocking Buffer and probed with Haptoglobin (Sigma H8636) antibodies followed by a corresponding IRDye secondary antibody (Licor Biosciences #926-32210 or #926-68021). The blot was developed using the Odyssey Infrared Imaging System (Licor Biosciences).

## Abbreviations

LTL: Lectin-directed Tandem Labeling; iTRAQ: Isobaric Tags For Relative and Absolute Quantitation; LC-MS/MS: Liquid chromatography–mass spectrometry; HPLC: High-performance liquid chromatography; SNA: *Sambucus Nigra* ; MS/MS: Mass Spectrometry/Mass Spectrometry; XIC: Extracted Ion Chromatogram; H/L: Heavy/Light; ACN: Acetonitrile; CaCl_2_: Calcium Chloride; NaH_2_PO_4_: Sodium Dihydrogen Phosphate; PMSF: PhenylMethaneSulfonyl Fluoride; AB: Ammonium Bicarbonate; FA: Formic Acid; TFA: Trifluoroacetic Acid; DTT: Dithiothreitol; IA: IodoAcetamide; MnCl_2_: Manganese Chloride; KCl: Potassium Chloride; NaCl: Sodium Chloride; LTQ: Linear Trap Quadrupole; HCD: Higher Energy Collisional Dissociation; PVDF: PolyVinylidene Fluoride.

## Competing interests

The authors declare that they have no competing interests.

## Authors’ contributions

VS: designed the methodology, participated in mass spectrometry, data analysis, verification of methodology, and drafted the manuscript. JH: participated in the sample preparation, performed western blot analyses and assisted in the preparation of the manuscript PS: participated in the sample preparation, mass spectrometry, data analysis and statistical analysis ZN: participated in sample preparation, mass spectrometry, and data analysis RP: managed the lab in which the study occurred, conceived of the study, help designed the study, and coordinated the entire project, as well as being substantially involved in the writing/editing of the manuscript. All authors read and approved the final manuscript.
